# Efficacy of school-based intervention programs in reducing overweight: A randomized trial

**DOI:** 10.3389/fnut.2022.1001934

**Published:** 2022-09-29

**Authors:** Santo Marsigliante, Vito Ciardo, Antonio Di Maglie, Giulia My, Antonella Muscella

**Affiliations:** ^1^Department of Biological and Environmental Science and Technologies (DiSTeBA), University of Salento, Lecce, Italy; ^2^Department of History, Society and Human Studies, University of Salento, Lecce, Italy

**Keywords:** children obesity, overweight, BMI, waist circumference, school-based intervention

## Abstract

Childhood obesity represents a serious public health problem and this study evaluates the effectiveness of a 6-month educational intervention on lifestyle, nutrient adequacy, and diet quality in the school setting in improving the knowledge and behavior of primary school children regarding correct eating habits. The strategy was implemented over a 6-month period and participants were randomly assigned to either the intervention group (*n* = 200) or the non-intervention group (control group, *n* = 197). Participants had a mean body mass index of 18.3 ± 2.7 kg/m^2^ and its variation in the intervention group (−2.7 ± 0.5 kg/m^2^) was significantly different from that in the control group (3.41 ± 0.8 kg/m^2^). In the experimental group, there were significant differences between the proportion of children who were overweight, underweight, normal weight, or obese before and after intervention (*p* < 0.05). The best results were seen in the female sex, and after the intervention, there were no more girls with obesity. Furthermore, there were significant waist circumference decrement effects in the intervention group compared to the control group (*p* < 0.05). Finally, many of the participating children acquired healthy eating habits. Therefore, the quantitative results obtained suggest that a school intervention program represents an effective strategy to prevent and improve the problem of childhood overweight and obesity.

## Introduction

Childhood overweight and obesity represent a major global health problem (1), and these conditions, if already present in childhood and adolescence, can certainly lead to serious chronic diseases (2, 3); when they persist in adulthood, cardiovascular disease, diabetes, and some cancers are their serious consequences (4–6).

Therefore, the monitoring of weight status in children is important for health disease prevention (7). The development of obesity in children is influenced by several modifiable behaviors and, in general, insufficient physical activity together with a high caloric intake are important factors contributing to the increase in obesity among young people (8, 9). In particular, dietary factors are importantly associated with childhood obesity (10) and the prevalence rates of diet-related diseases, such as obesity and diabetes in children and adolescents, push for the right priority to healthy diets (8, 9, 11). Many children, instead of a diet rich in fruits and vegetables, choose snacks with saturated trans fats and sugar (12), which lead to a higher energy intake (13) and consequently to overweight/obesity (12, 14, 15). The assumption of harmful behaviors, such as eating unhealthy foods, during the time spent in a sedentary lifestyle, has been shown to exacerbate the capacity to maintain an adequate body weight in children by creating a vicious cycle (16, 17). Both physical inactivity and sedentary/unhealthy behaviors increase the risk of being overweight and obese in childhood (18–20). Hence, the role of diet and physical activity in children has been investigated in previous studies (21, 22). Based on the above, Tabacchi et al. (21) suggested that raising children in an environment where both motor and cognitive skills are encouraged can improve food literacy and, furthermore, enhance school achievement.

Several lifestyle interventions were able to lower anthropometric and cardiometabolic risk factors (20, 23–26) and increase the body image satisfaction (27), but few interventions have considered changes in diet quality (28, 29). Unfortunately, children are frequently reluctant to comply with exhortation weight-related behaviors and response to behavioral treatment is generally limited and confined to the short term (30–32).

Obviously, as children spend a lot of their time in school, it is important to implement school programs aimed at defining and maintaining a healthy lifestyle (33–35), thus increasing the cognitive functioning and mental health and wellbeing (36). Therefore, the aim of our study was to evaluate the effect of a 6-month intervention on lifestyle, nutrient adequacy, and diet quality in children attending schools in Southern Italy.

## Methods

### Participants

To understand the effects of promoting nutrition adequacy, a sample of 398 children was selected from different schools. These schools are located in two cities with similar socioeconomic status and have not previously participated in health promotion programs. Schools were secondary-level public schools, with attendance from 8:00 to 13:00.

The sample was composed of 194 boys and 204 girls, who were allocated to a control group and a group participating in an intervention (*n* = 200 and *n* = 198, respectively). All children were healthy and free of any disability or musculoskeletal, cardiological, neurological, or respiratory diseases or dysfunctions. Before the inclusion of the children in our program, a parent or legal representative of each child signed informed consent. The study was conducted in accordance with the Helsinki Declaration and the European Union recommendations for Good Clinical Practice (document 111/3976/88, July 1990). The University's Research Ethics Committee approved the study.

**Figure 1:**
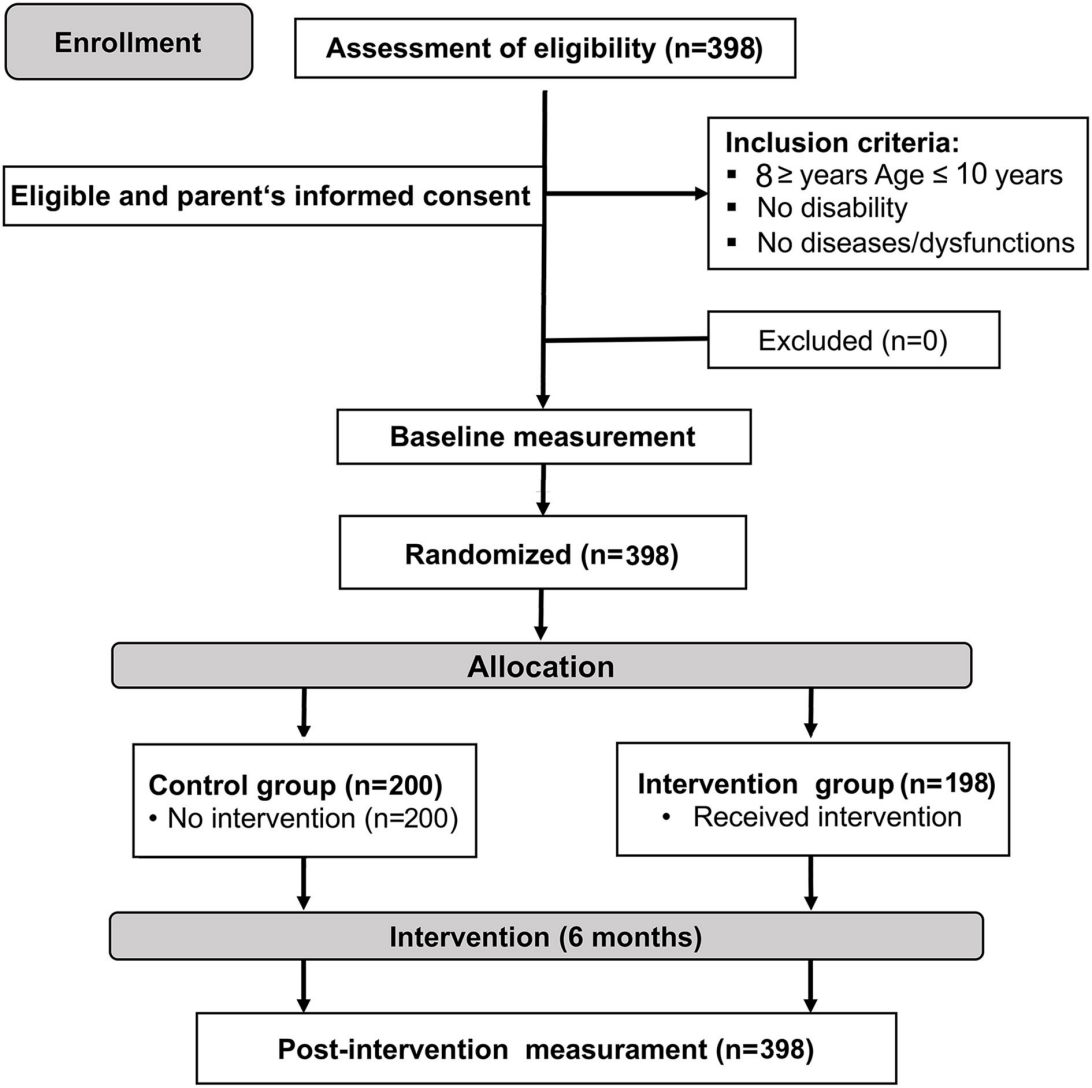
Scheme of the participants in the study.

A randomized controlled trial study design, which is double-blinded, was employed; the design is shown in Figure 1.

### Food education program

Before the start of the project, all teachers and parents in the intervention schools received on-site training to provide them with general information on the nature and significance of the intervention and to support their role in educating the children. The intervention conducted aimed at improving sugar-containing beverages consumption, high-caloric snack consumption, and promoting the consumption of vegetables and fruit. The goal of this program is to decrease energy intake while improving food quality. Therefore, information on portion control was provided with no indication of kilocalories or grams of food consumption. Food education and a healthy lifestyle (e.g., food choices, food labels, the five meals, consumption of fruits and vegetables, and sleep quality) were discussed with the active involvement of everyone.

The educational intervention covered 12 lessons for the subject's biology and alimentation implemented by classroom teachers. The first part (six lessons) aimed at increasing awareness and information regarding energy balance-related behaviors, with supporting materials, such as a pocket-sized diary, to monitor own behavior. The second part (six lessons) aimed at facilitation of choice to improve one of the behaviors, setting personal goals, identifying barriers, improving self-efficacy, and evaluating the change process. In this way, the children and families understand how to organize their weekly meal planning without detailed prescriptions. The control schools followed their regular curriculum.

### Measures

All measures were recorded at baseline (T0) and 6 months from baseline (T1) at the completion of the intervention.

Body weight and height were ascertained, in duplicate, with standard techniques. Waist circumference was measured, in duplicate, at the iliac crest, and at mid-respiration using a non-elastic measuring tape. The ratio between waist circumference and height (WHR) was calculated. The cut-off used to represent CV risk for WHR was 0.500 (37).

For children and adolescents, the Center for Disease Control and Prevention defines overweight as a body mass index (BMI: weight in kilograms divided by height in meters squared) between the 85th and 95th percentiles and obesity as a BMI at or above the 95th percentile for sex and age (38).

The parents completed structured no validated questionnaires, which included the eating habits of their children (food frequency questionnaire) and their familiarity with certain food items. This questionnaire was administered twice to the parents of the children, the first time at the beginning of the study and the second time at the end of the intervention; in both cases, 48 h were given for completion. The data collected with the questionnaire were analyzed by extrapolating the frequency of each item considered in the proposed survey. Descriptive data was represented by the percentage.

### Statistical analysis

Means and standard deviations were described for the total sample and for both the participants and control groups. All data have been tested for normality using the Kolmogorov–Smirnov test. To identify differences between groups, the *t*-test has been performed. For the association of different items of the questionnaire, a chi-square test was performed. The significance level was set to *p* < 0.05.

## Results

The sample consisted of 397 children who were randomly divided into two groups, the intervention group, and the control group. Table 1 shows the characteristics of the 197 children in the intervention group and the 200 children who made up the control group. The mean age was 9.5 ± 0.5 years for both sexes.

**Table 1:** Physical characteristics of children (Mean ± SD).

**Characteristic (Participant group)**	**All (*n* = 198)**	**Boys (*n* = 94)**	**Girls (*n* = 104)**
Age, y	9.1 ± 0.5	9.3 ± 0.7	9.2 ± 0.6
Height, cm	134.9 ± 11.5	136.8 ± 12.4*	133.5 ± 10.8*
Weight, Kg	34.4 ± 10.2	36.6 ± 10.8*	32.6 ± 9.4*
BMI	18.4 ± 2.6	19.1 ± 2.7	17.9 ± 2.4*
BMI, percentile	68.3 ± 24.6	74.3 ± 21.3	62.3 ± 25.3*
Waistline	58.7 ± 4.6	61.3 ± 5.1	56.8 ± 1.4*
**Characteristic** **(Non-participant group)**	**All** **(*****n*** **= 200)**	**Boys** **(*****n*** **= 100)**	**Girls** **(*****n*** **= 100)**
Age, y	9.1 ± 0.7	9.2 ± 0.5	9.3 ± 0.5
Height, cm	134.8 ± 9.8	135.9 ± 12.4	133.2 ± 9.8*
Weight, Kg	34.5 ± 9.9	35.8 ± 10.3	33.1 ± 10.2*
BMI	18.1 ± 2.7	19.7 ± 2.9	18.1 ± 2.4*
BMI, percentile	68.5 ± 23.8	73.9 ± 25.6	62.9 ± 25.9*
Waistline	58.9 ± 4.7	60.9 ± 5.4	56.7 ± 2.8*

BMI, body mass index (calculated as weight in kilograms divided by height in meters squared). WHR, waist-to-height ratio; *p < 0.05 between girls and boys, by t-test.

Coherent with the random assignment of children to the intervention and control groups, no significant differences were observed between these groups in terms of average weight, height, BMI, and proportion of overweight or obese children (*p* > 0.05; Table 1).

Statistically significant differences between the sexes were noted, being the boys taller and heavier than the girls. Boys also have a larger waistline (*p* < 0.05; Table 1).

**Figure 2:**
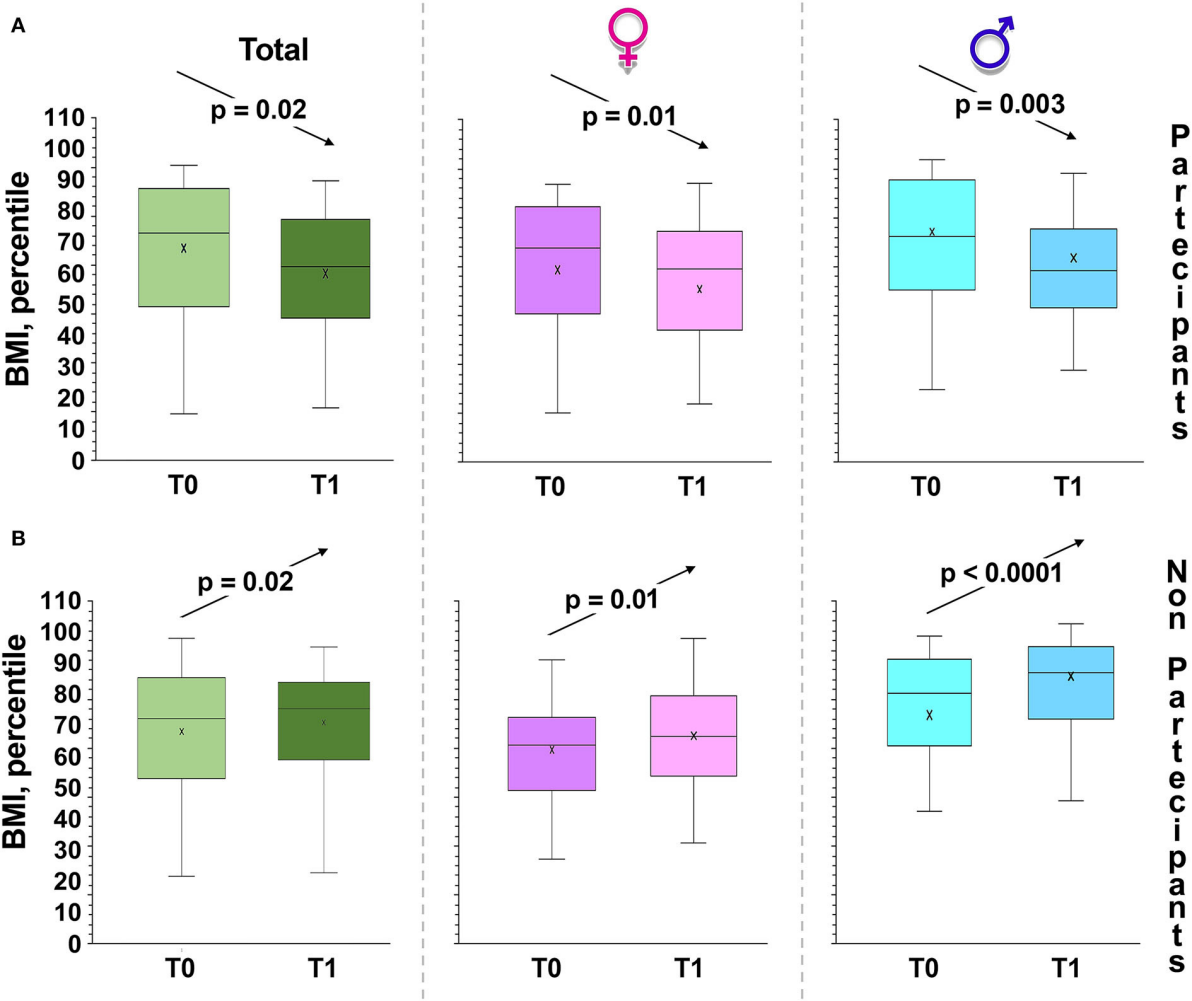
Changes in BMI, percentile over time. Box and whiskers representation of body mass index (BMI: weight in kilograms divided by height in meters squared) of intervention group **(A)** and control children **(B)**, recorded at baseline (T0), at 6 months from baseline (T1, at the completion of the intervention). The sample was also divided between girls and boys. In this representation, the central box covers the middle 50% of the data values, between the upper and lower quartiles. The bars extend out to the extremes, while the central line is at the median. *p*-values were obtained by Student's paired *t*-test.

Interestingly, while BMI increases in the untreated group, it significantly decreases in the intervention group (Figure 2).

As regards the detection of percentiles in the different age groups, we have highlighted that overweight and obesity in children increased with age (Figure 2). In fact, before our educative intervention, 18.5% of the children were underweight. We also highlighted significant differences between the proportions of overweight, underweight, normal weight, or obese children before and after intervention in the experimental group (Figure 2; χ^2^, *p* = 0.0001).

**Figure 3:**
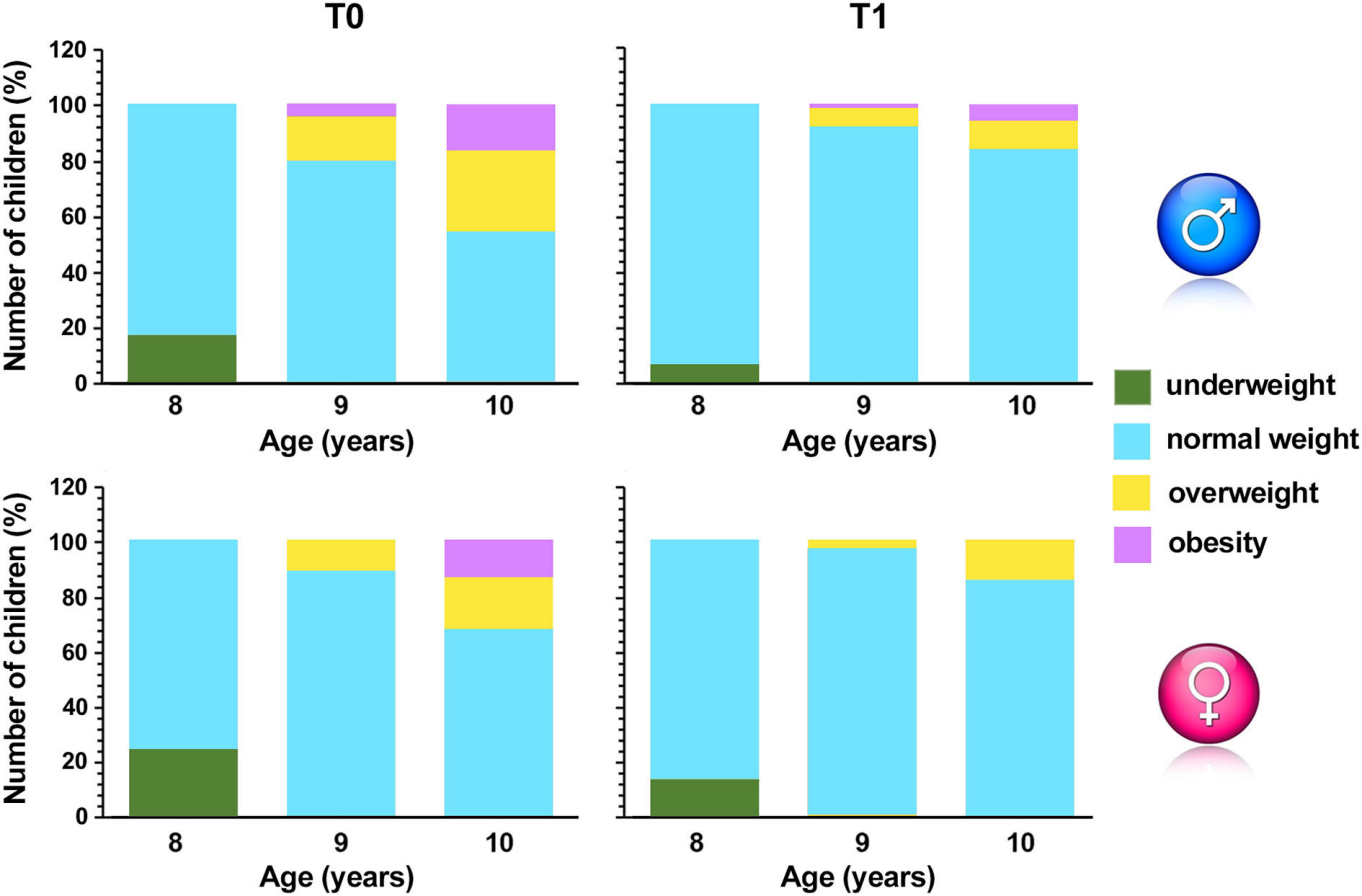
Percentage of overweight, underweight, normal weight or with obesity children pre (T0) and post (T1) intervention. The sample was also divided between girls and boys and different age groups.

The most striking results were obtained in females, since girls with obesity were no longer detectable after intervention (Figure 3).

**Figure 4:**
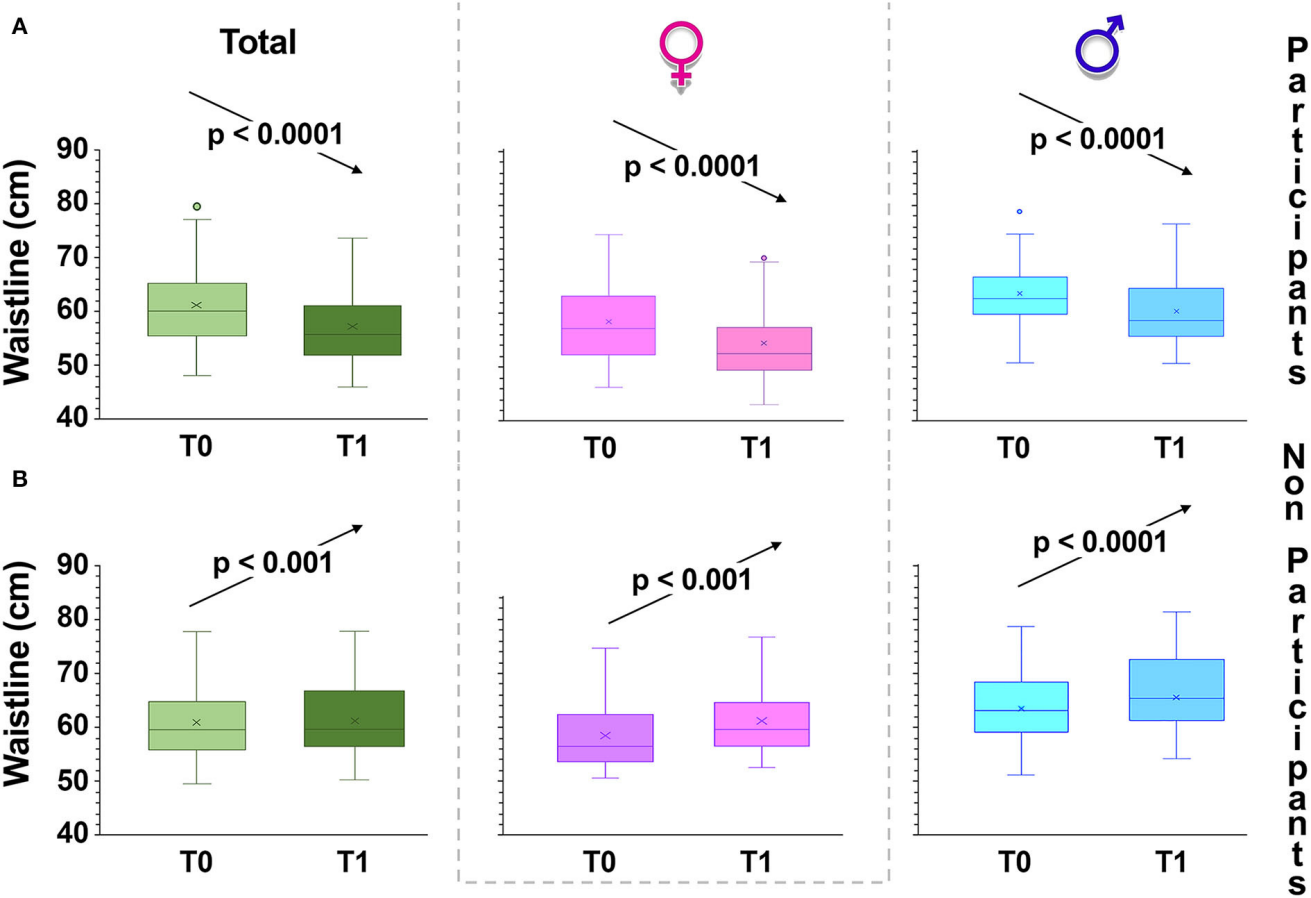
Changes in waist circumference over time. Box and whiskers representation of waist circumference of intervention group **(A)** and control children **(B)**, recorded at baseline (T0) and 6 months from baseline (T1, at the completion of the intervention). The sample was also divided according to gender. In this representation, the central box covers the middle 50% of the data values, between the upper and lower quartiles. The bars extend out to the extremes, while the central line is at the median. Those values which are beyond 1.5 times the interquartile range beyond the central box are plotted as individual points. *p*-values were obtained by Student's paired *t*-test.

Another indicator of obesity was also measured; in the experimental group, the waist circumference substantially declined (*p* < 0.001) (Figure 4A). In contrast, in the group that is not participating in the intervention, waist circumference significantly increases, especially in boys (Figure 4B).

WHR also includes height and is, in normal-weight children as well, an important indicator of excess upper body fat (37). Hence, WHR allows to identify children at risk of developing metabolic complications (39), with a cut-off value greater than 0.500 (37, 40). In T1, mean WHR decreased from 0.43 ± 0.06 to 0.41 ± 0.03 (*p* < 0.001) (males: 0.45 ± 0.013; to 0.42 ± 0.01; *p* < 0.01; females from 0.42 ± 0.03 to 0.41 ± 0.03; *p* < 0.05) in the participating group, while it remained unchanged in the control group. Furthermore, the number of children who had a ratio >0.50 in T0 also decreased significantly (χ^2^, *p* = 3.46·10^−5^).

### Questionnaire results

The data collected in T0 and T1 using the questionnaires were analyzed by extrapolating the frequency of each item considered in the proposed survey and presented as a percentage (Tables 2, 3). Our questionnaires show that only 42% of our population (*n* = 398) ate breakfast regularly. A more equal distribution of the mid-morning meal was observed, with 32.6% “yes,” 35.6% “sometimes,” and 31.8% “no” (Table 2). An afternoon snack was consumed by 79% of the children (Table 2). The first choice was sweets for both midmorning and afternoon snack (48.5 and 52.25%, respectively), among biscuits, chips, and cold cuts (Table 2).

**Table 2:** Changes in dietary parameters after lifestyle intervention in children (*p* by chi-square test).

	**Participant group** **(*****n*** = **198)**			**Non-participant group** **(*****n*** = **200)**		
	**T0**	**T1**	***p***	**T0**	**T1**	***p***
	**Do you have breakfast?**					
Yes	42.5%	66.5%		41.8%	42%	
Sometime	32%	28%	3.5 ·10^−6^	31.5%	33%	0.98
No	25.5%	5.5%		26.7%	25%	
	**Do you eat a snack in the morning?**					
Yes	32.7%	70.5%		32.5%	33.5 %	
Sometime	36.8%	16.5%	0.02	34.5%	38.5%	0.53
No	30.5%	13%		33%	28	
	**Do you eat a snack in the afternoon?**					
Yes	79.5%	92%		82%	80%	
Sometime	17%	5.5%	0.007	15.5%	17.5%	0.85
No	3.5%	2.5%		2.5%	2.5%	
	**What do you eat in the morning?**					
Fruit	18%	63%		18.5%	20%	
Sweets	47.5%	6.5%	3·10^−32^	49.5%	48.5%	0.94
Cips	2%	1%		4%	3%	
Cold cuts	32.5%	28.5%		28%	28.5%	
	**What do you eat in the afternoon?**					
Fruit	20.5%	73%		19.5	18.5	
Sweets	52%	12.5%	7·10^−37^	52.5	53.5	0.99
Cips	7.5%	2%		6.5	6	
Cold cuts	20%	12.5%		21.5	22	

**Table 3:** Change in diet quality after lifestyle intervention in children.

**Food**	**No**.	**Participant group** **(*****n*** = **198)**			**Non-participant group** **(*****n*** = **200)**		
		**T0**	**T1**	***p***	**T0**	**T1**	***p***
Fruits/day	0	12.5%	2.5%		10%	9%	
	1	37%	8.5%		41.5%	38%	0.72
	2	28%	10.5%	1.3·10^−6^	31%	28.5%	
	3	13.5%	66		10.5%	14.5%	
	≧ 4	9%	12.5		7%	10%	
Vegetables/day	0	20%	4%		20%	18.5%	
	1	27%	9.5%		28%	27%	
	2	22%	40%	0.0002	21.5%	22%	0.51
	3	20%	30.5%		20.5%	21.5%	
	≧ 4	11%	16%		10%	11%	
Sweet/day	0	5%	5%		12%	12%	
	1	41.5%	45%		35%	31.5%	
	2	13.5%	31%	0.0002	13%	17.5%	0.80
	3	26%	12.5%		26%	26%	
	≧ 4	14%	6.5%		14%	13%	
Sweetened drinks(coca-cola, tea, juices)	0	6%	23%		6.5%	8%	
	1	43%	49%		43.5%	44.5%	
	2	25.5%	15%	1.26·10^−6^	25.5%	24.5%	0.64
	3	13%	7.5%		12%	11.5%	
	≧ 4	12.5%	5.5%		12.5%	11.5%	

p by chi-square test.

Since one of the lifestyle-related risk factors of being overweight is skipping breakfast (41, 42), we have considered this risk factor as an objective of our preventive program. In T0 the children who never had breakfast were 25.5% of the participating group and after the intervention (T1) this percentage dropped to 5.5%. Consistently, the children who reported having breakfast every day in T0 were 42.5% and this had increased to 66.5% in T1 (χ^2^ = 33.156, *p* < 0.0001). The children in the control group maintained the same percentages (Table 2).

In T0, while as mentioned earlier there was an equal distribution of the mid-morning snack, the afternoon snack was consumed by 80.8% of children (Table 2). In T1, the children of the experimental group who eat a snack in midmorning and, in the afternoon, increased further (Table 2; χ^2^ = 5.302, *p* = 0.020, χ^2^ = 9.453, *p* = 0.007, respectively).

Among fruit, chips, and cold cuts, the first choice for a midmorning (48%) or afternoon snack (52%) was sweets (Table 2). In T1, children in the experimental group preferred to eat fruit for both snacks (Table 2; χ^2^ = 10.805, *p* < 0.0001, χ^2^ = 16.133, *p* < 0.0001, respectively).

An important aspect of the food education program was to consume healthy and complete meals consisting of a first course, second course, vegetables, and fruits. From the data analysis, it emerged that 80% of the population consumes vegetables almost every day; and even 20% never eat vegetables (Table 3). After our intervention, the consumption of vegetables increased significantly among the children participating in the program (Table 3; χ^2^ = 9.311, *p* = 0.0002). Although experts advise against them, 12.5% of children drink sugar-added and non-nutritious drinks every day (Table 3). In T1 this percentage decreases significantly, in the experimental group; in addition, 23% of children no longer drink sugar-added drinks (Table 3; *p* = 0.0001).

## Discussion

As the number of overweight and obese children has increased in recent decades, the need to support healthy nutrition and physical activity in children has never been more urgent.

Thus, the present study aims to investigate the effectiveness of an education program, regarding correct eating habits on children's weight status.

In Europe, higher levels of children overweight and obesity have been reported in southern countries (42), particularly for those children growing up in low-income families (43). This socio-economic variability of childhood obesity and its relationships with lifestyle has also been reported in Italy (44), with prevalence higher in southern regions where socio-economic conditions are worse than those in northern regions (45). Following the recommendations of the WHO European Ministerial Conference on the fight against obesity (46), the Italian Minister of Health financed the National Nutritional Surveillance System called “OKkio alla SALUTE” (47, 48), which made it possible to make comparisons between Italian countries within the European Region (49).

In our study, we find that the number of overweight students was about 20% (22% of males and 18.5% of females), a percentage which is in line with what was previously reported for our region (49). More precisely, the percentages of 11-year-old students who were overweight were: males 14.5, 21.3, and 28.5%; females 11.6, 13.5, and 21.1% in the north, center, and south of the country, respectively (47–49). The percentage of obesity reported here is also in agreement with Lauria's study (49): 12% and 6% for males and females, respectively. It is interesting to note that the percentages of children with overweight and obesity increased significantly from 8 to 10 years.

Since this study was conducted from 2017 to 2018, these higher rates of obesity are not caused by the COVID-19 pandemic-dependent lockdown but were due to pre-existing socio-economic conditions and other health indicators.

In childhood obesity, the inadequate intake of fruit and vegetables and the consumption of too many high-calorie snacks play a very important role, with 41% of total calories coming from chips, chocolate bars, soft drinks, fruit drinks, sugars, syrups, preserves, fats, and oils (50).

As known, family, friends, school, marketing, and the media influence children's food choices. To prevent childhood obesity, it is useful to combine family and school programs and nutrition education with adequate physical activity (21). Habits that protect against childhood obesity include having breakfast and eating family meals and being physically active. In this context, we examined the effects of a 6-month pilot intervention on students from schools located in two small towns with similar socio-economic conditions. This intervention had the aim of educating on a healthy diet according to the dictates of the Mediterranean diet (especially breakfast, mid-morning snacks, and fruit-based afternoon snack; vegetables for lunch and dinner; drinking water instead of sugary drinks) and to carry out at least 60 min of physical activity per day.

An important source of primary nutrients in the diet of both adults and young people is breakfast (51–53), which has been shown to be able to produce many benefits (53). Daily breakfast consumption also has a protective action against obesity and overweight (54) with a clear inverse association with body fat (55). Indeed, obese children have been reported to skip breakfast (42, 55, 56) and had a high risk of chronic diseases, such as type 2 diabetes (57), dyslipidemia, or cardiovascular disease (54). Finally, breakfast consumption also has a positive effect on children's cognitive performance (58). Unfortunately, in agreement with previous studies (59, 60), only 42.5% of children said they had breakfast every day. In T1, however, this percentage increased to 66.5%.

We suggested that the children eat five meals a day, introducing the consumption of mid-morning and afternoon snacks into their diet. Some healthy eating habits were acquired in most cases: eating fruit as a midmorning and afternoon snacks and avoiding the consumption of cold cuts and sweet foods. It is worth noting that 63% and 73% of children chose fruits for morning and afternoon snacks respectively, and 78.5 ate fruits more than three times a day. These results showed better percentages than the international data of “NCD Risk Factor Collaboration (NCD-RisC) (1),” in which 35.4% of adolescents (aged 11–15) consumed at least one portion of fruit daily, and 27.3% ate vegetables at least once a day (1). On the contrary, 18% of adults treated in the Obesity Unit of Hospital of Torino reported a decrease in fruits and vegetables consumption during the COVID lockdown (61).

Therefore, our intervention gave an encouraging result within a scenario in which foods rich in fibers have been replaced by products rich in fat and sugars, with a high level of processing (62). Most of these products contain additives to make them durable and hyper-palatable. However, they have very low nutritional quality, and their consumption tends to limit the consumption of unprocessed or minimally processed foods (63). Consumption of ultra-processed foods has been pointed out as a risk factor for increasing obesity, as measured by BMI, among children, adolescents, and adults (41).

The present study has some limitations, primarily the different ages of the participants. However, the weight loss was substantial as well some lifestyle indicators have undergone significant improvements. Another limitation of the present study is the lack of data on the socio-economic, cultural, and educational levels of parents. Larger studies with long-term follow-up are therefore needed to corroborate our findings.

## Conclusion

We evaluated the effect of a 6-month-long intervention on lifestyle and diet quality in schools in Southern Italy. The children and adolescents participating in the study achieved a reduction in BMI, waist circumference, and better adherence to nutritional recommendations assessed by the diet quality indices. The school intervention program represents an effective strategy for preventing the problem of childhood overweight and obesity.

## Data availability statement

The datasets presented in this article are not readily available because of participant privacy. Requests to access the datasets should be directed to antonella.muscella@unisalento.it.

## Ethics statement

The studies involving human participants were reviewed and approved by IRB of Department of Biological and Environmental Science and Technologies (DiSTeBA), University of Salento, Via Prov. le Lecce-Monteroni, 73100 Lecce, Italy. Written informed consent to participate in this study was provided by the participants' legal guardian/next of kin.

## Author contributions

AM, VC, and SM: conceptualization. ADM, VC, AM, and SM: data curation. VC, GM, and ADM: investigation. AM and SM: project administration and original draft. AM: supervision and writing. SM: writing—review and editing. All authors contributed to the article and approved the submitted version.

## Conflict of interest

The authors declare that the research was conducted in the absence of any commercial or financial relationships that could be construed as a potential conflict of interest.

## Publisher's note

All claims expressed in this article are solely those of the authors and do not necessarily represent those of their affiliated organizations, or those of the publisher, the editors and the reviewers. Any product that may be evaluated in this article, or claim that may be made by its manufacturer, is not guaranteed or endorsed by the publisher.
